# Surgical staging identified false HPV-negative cases in a large series of invasive cervical cancers

**DOI:** 10.1016/j.pvr.2017.10.003

**Published:** 2017-10-19

**Authors:** Karl Ulrich Petry, Clemens Liebrich, Alexander Luyten, Martina Zander, Thomas Iftner

**Affiliations:** a Department of Obstetrics and Gynecology, Klinikum Wolfsburg, Wolfsburg, Germany; b Institute of Pathology, Klinikum Wolfsburg, Wolfsburg, Germany; c Institute of Experimental Virology, University of Tübingen, Germany

**Keywords:** Cervical cancer, HPV-negative, Adenocarcinoma, Diagnosis

## Abstract

**Objective:**

We examined a large series of biopsy-proven invasive cervical cancers with surgical staging and HPV re-testing to estimate the relevance of HPV-negative cervical cancers in a Caucasian population.

**Methods:**

We prospectively collected smears from 371 patients with a biopsy-proven diagnosis of cervical cancer for HC2 testing of high-risk HPV (HR-HPV). In HC2-negative cases, smears and paraffin embedded tissue blocks underwent additional HPV genotyping.

**Results:**

HC2 tests showed 31/371 cases (8.8%) had negative findings. Surgical staging showed that 21/31 HC2-negative cases (68%) were not cervical cancer. Overall, 340/350 cases of primary cervical cancer confirmed by surgical staging tested HC2 positive (97.2%). Non-high-risk HPV subtypes were detected in five cases (one HPV-53, one HPV-70, and three HPV-73) and high-risk subtypes in four patients with HC2-negative cervical cancer (two HPV 16 and two HPV-18). The remaining case, a primary undifferentiated carcinoma of the uterine cervix, tested negative for HPV-DNA with all tests.

**Conclusions:**

The main explanation for HPV-negative cervical cancer was a false diagnosis, followed by cancers associated with non-HR-HPV types, and false-negative HR-HPV results. Truly HPV negative seem to be very rare in Caucasian populations. Retrospective analyses without surgical staging may overestimate the proportion of HPV negative cervical cancers.

## 1 Introduction

Primary screening for human papilloma virus (HPV) is more sensitive than cytology-based screening to detect high-grade neoplasia and more efficient in the prevention of cervical cancer [1], [2]. Based on such compelling evidence, European, Australian, and US American guidelines now recommend HPV screening as the preferred concept to prevent cervical cancer among women who are 30 years or older [3], [4], [5]. Studies have shown that, generally, fewer than 10% of cases of invasive cervical cancer are classified as ‘HPV negative’ using standard tests for high-risk subtypes [6], [7], [8], [9], [10], [11], [12], [13]. Previously, data from the ATHENA study was analyzed to identify ‘true’ HPV-negative cervical lesions using samples initially screened using a cobas HPV Test, which identifies 14 high-risk HPV types (16, 18, 31, 33, 35, 39, 45, 51, 52, 56, 58, 59, 66, and 68), compared with Linear Array and Amplicor testing and blinded histopathology review [14]. Overall not a single CIN3 lesion could be detected that was truly HPV negative among 305 CIN3 cases. Careful examination of all cobas HPV-negative CIN3 lesions in the ATHENA trial demonstrated that false diagnosis, CIN3 associated with HPV-types not considered to be high-risk types, and high-risk HPV subtypes missed by the initial cobas HPV test were possible explanations for these negative findings [14].

Some recent publications have suggested that HPV testing may miss a significant proportion of ‘HPV-negative’ invasive cervical cancers, especially certain types of adenocarcinoma [15], [16], [17]. For example, the authors of a US-based retrospective study suggested that up to 19% of women with cancer may be given a misleading test result using HPV-only testing and recommended co-testing with HPV and Pap cytology [17]; however, these findings have been challenged and concerns raised that co-testing may lead to substantially more false-positive cases, while not improving the efficiency of cervical cancer prevention [18].

CIN3 is considered the direct precursor of cervical cancer with a high, long-term risk of malignant progression [19], [20]. The detection and treatment of CIN3 in screening programs is the main mechanism leading to a significant reduction in cervical cancer incidence rates in industrial countries [21]; therefore, and in the light of analyses from the ATHENA study, HPV-negative cervical cancer would seem to be an unpreventable disease in view of the virtual non-existence of HPV-negative CIN3 [14], [22]. In contrast, the existence of HPV-negative cervical cancers is well documented [10], [11], [17], [23], [24] and a rare subset of cervical adenocarcinoma not associated with HPV infection has been described [15], [25]. However, the true incidence of HPV-negative invasive cervical cancers is likely to be overestimated in studies based on a single HPV test [18]. We hypothesized that the incidence of true HPV-negative invasive cervical cancers is close to zero in patients evaluated using multiple HPV tests and additional information obtained from surgical staging procedures. The objective of this study was to determine the incidence of true HPV-negative invasive cervical cancer among patients who were surgically staged; however, the present study does not consider the specific benefits and risks of surgical staging in this patient population.

## 2 Patients and methods

Women with primary cervical cancer diagnosed by biopsy or histological assessment in our colposcopy clinic between April 1999 and March 2015 were eligible for inclusion in this study. Patients with cervical cancer who did not undergo surgery or HC2 testing were not eligible. The Ethics Committee of Lower Saxony was consulted, but stated formal ethical approval was not required because the study was based only on anonymous routine clinical data.

Cervical swabs using a brush were taken from all participants. HPV testing was done routinely with hybrid capture 2 (HC2) according to the standard protocol recommended by the manufacturer (Qiagen, Hilden, Germany). HC2 detects high-risk HPV types 16, 18, 31, 33, 35, 39, 45, 51, 52, 56, 58, 59 and 68. The threshold for a positive HC2 result was 1 RLU (relative light unit).

All participants underwent surgical staging in our department. In stage Ia1 cases, excision of the transformation zone with clear margins was considered sufficient, while stage Ia2 cases underwent at least excisional treatment and sentinel lymph node biopsy or hysterectomy with complete pelvic lymph-node extirpation. Stage Ib-IIa cases were typically treated by radical hysterectomy with lymphadenectomy either by laparotomy or laparoscopy, and more advanced stages underwent exploratory laparotomy/laparoscopy with exploration of the abdominal cavity and removal of pelvic and para-aortal lymph nodes.

A diagnosis of primary cervical cancer was based on surgical staging with exclusion of malignancies of other origin. If necessary, immunohistochemistry was used to confirm sarcoma, lymphoma, or cancers of other origin. Distinguishing between endometrial cancer and endometrioid cervical cancer is difficult and, in advanced stages, sometimes arbitrary; therefore, for this analysis only, endometrioid cancers with exclusive or dominant infiltration of the uterine cervix that spared at least parts of the uterine cavity were defined as cervical cancers. Patients were excluded from further analysis if a positive diagnosis of cervical cancer was not confirmed by surgical staging.

Primary cervical cancers that were confirmed by surgical staging but tested negative for HR-HPV by HC2 underwent further HPV-testing. Paraffin embedded tissue blocks were deparaffinized and DNA was extracted automatically using the QIAsymphony DSP DNA Mini Kit (Qiagen, Hilden, Germany). SPF10 HPV genotyping was performed using the INNO-LiPA HPV Genotyping Extra® test (abbreviated as ‘LiPA’; Fujirebio LiPA Extra), which identifies 20 HPV genotypes classified as Group 1, 2A and 2B carcinogens (16, 18, 26, 31, 33, 35, 39, 45, 51, 52, 53, 56, 58, 59, 66, 68, 69, 70, 73, 82) and eight low-risk HPV or intermediate-risk genotypes (6, 11, 40, 43, 44, 54, 71, 74). LiPA Extra is a line blot assay based on SPF-10-PCR as described previously [26]. Strips were scanned and analyzed automatically with a flatbed scanner and the LiRAS software (Fujirebio, Belgium). In cases with negative LiPA results, we performed a nested PCR with the PGMY09/11 primer pairs first and used the amplicon for the second PCR using the GP5+/6+ primer sets (abbreviated as ‘nested PCR’). Amplicons were then sequenced using the GP6+ primer and the respective HPV type was identified by comparing the sequence with the NCBI data base using BLAST [27].

The study endpoints for patients with a negative HC2 test and confirmation of invasive cervical cancer after surgical staging were histology (squamous cell carcinoma, adenocarcinoma, or other), HPV status on additional tests, and specific HPV subtype. The objective of the study was to determine whether any patients had a definitive HPV-negative invasive cervical carcinoma. All data are reported descriptively for individual patients.

## 3 Results

**Fig. 1:**
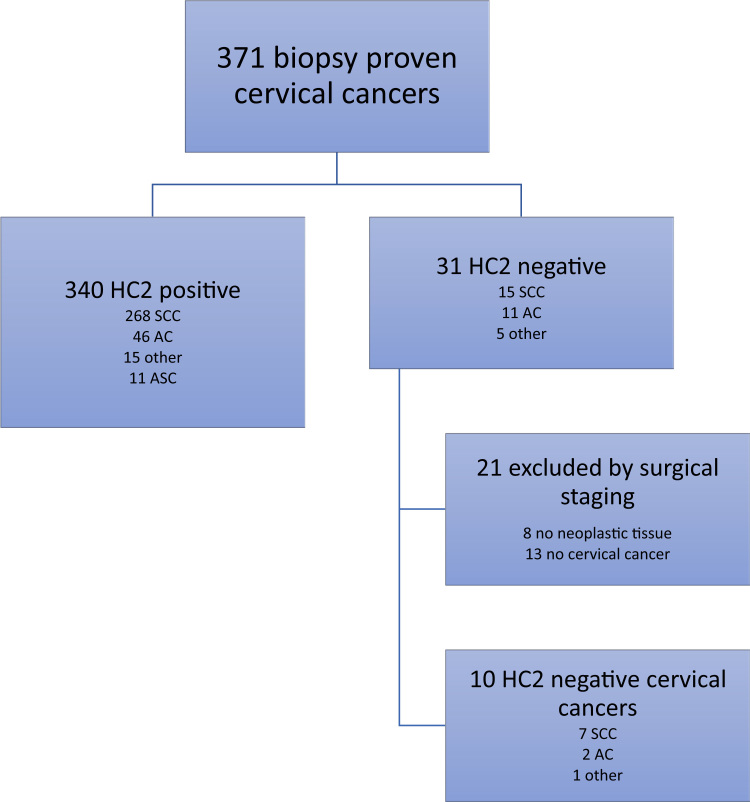
Flow of patients included in the study. AC = adenocarcinoma, ASC = adenosquamous carcinoma, HC2 = hybrid capture 2; SCC = squamous cell carcinoma.

The analysis included 371 cases with an initial diagnosis of primary cervical cancer and a HC2 test result (Fig. 1). The median age of patients was 48.7 years (range 24–87 years). HC2 showed that 340 cases (91.2%) tested positive for HR-HPV, while the remaining 31 cases had negative HC2 results. In eight of the 31 HC2-negative cases, cone biopsy specimens initially showed margin involvement of stage I cervical cancers prior to referral; HC2 sampling was done after conization, but the final (radical) hysterectomy specimens did not show any residual disease and these cases were excluded from further analyses.

Surgical staging of the remaining 23 HC2-negative cases confirmed the diagnosis of primary cervical cancers in 10 cases, while 13 cases were reclassified as malignancies of other origins. Specifically, two cases with cervical cancer, diagnosed at another center prior to referral, were reclassified as B-cell lymphomas on the final specimens.

Overall 340 of 350 confirmed primary cervical cancers were HPV positive and 10 were HPV negative. Additional HPV tests identified HPV infection in 9 of 10 cases (Table 1), comprising two cases of HPV-16, two cases of HPV-18, and five cases of other HPV types not considered as high-risk HPV (three of HPV-73, and one each of HPV-53 and HPV-70).

**Table 1:** Details of HC2-negative cancers, confirmed as primary cervical cancers by surgical staging.

**Age at diagnosis**	**Histology**	**HPV genotyping**		**Summary**
		**LiPA**	**Nested PCR**	
56 years	SCC	HPV 53	NA	Non-HR-HPV positive
65 years	SCC	HPV 73	NA	Non-HR-HPV positive
39 years	SCC	HPV 73	NA	Non-HR-HPV positive
52 years	SCC	HPV 73	NA	Non-HR-HPV positive
77 years	SCC	HPV 70	NA	Non-HR-HPV positive
74 years	SCC	HPV 16	NA	HR-HPV positive (false-negative HC2)
66 years	Undifferentiated	negative	HPV 16	HR-HPV positive (false-negative HC2)
72 years	Adenocarcinoma (clear cell)	HPV 18	NA	HR-HPV positive (false-negative HC2)
44 years	Adenocarcinoma (NOS)	negative	HPV 18	HR-HPV positive (false-negative HC2)
65 years	Undifferentiated	negative	negative	True HPV negative

HC2 = Hybrid Capture 2; LiPA = INNO-LiPA HPV Genotyping Extra^®^ test; NA = not applicable; NOS = not otherwise specified; PCR = polymerase chain reaction; SCC = squamous cell carcinoma.

Histologic assessment of 350 confirmed primary cervical cancers showed that 275 (78.6%) were classified as squamous cell carcinoma (SCC), 48 (13.7%) as adenocarcinoma (AC), 11 (3.1%) as adenosquamous carcinoma (ASC), and 16 (4.6%) as other types, including neuroendocrine small cell cancer (n = 4), undifferentiated cancer, and mixed types. Cases of adenocarcinoma were classified mainly as not otherwise specified (NOS), mucinous, or endocervical type, while there were only a few cases of clear cell adenocarcinoma (n = 2), villoglandular (n = 2), endometrioid (n = 3), or mucinous intestinal types (n = 1). SCC accounted for 7 of 10 cases of HC2-negative primary cervical cancer (Table 1). The single case of true HPV-negative primary cervical cancer was classified as undifferentiated histology.

## 4 Discussion

The aim of this study was to characterize the histopathology of women with putative HPV-negative cervical cancer using highly sensitive HPV testing and rigorous histological assessment following surgical staging. The results showed that true HPV-negative primary cervical cancers are very rare.

Our results are in accordance with The Cancer Genome Atlas Research Network (CGARN) ‘Integrated Genomic and Molecular Characterization of Cervical Cancer Study’, which used next-generation sequencing to characterize primary cervical cancers [28]. The CGARN study found 95% of primary cervical cancers were HPV-positive and 5% HPV-negative. Importantly, the study also identified a unique set of endometrial-like cervical cancers, which were predominantly HPV-negative cancers [28]. In agreement with our study, this finding suggests that ‘true’ cervical cancers are HPV-positive and even distinct histological types share similar clinical and molecular properties, whereas HPV-negative cancers are pathologically different. This distinction is particularly relevant in the debate about whether endometrioid cervical cancers or cervical extension of endometrium cancers represent cancers of the endometrium or a subgroup of cervical cancer [29]. The CGARN study shows that such cancers are identical to endometrium cancer and should be treated accordingly.

Previous studies of HPV prevalence among patients with cervical cancer have generally reported higher rates of HPV-negative tumors than we showed in this study [29], [30], [31], [32], [33]. The majority of HPV negative cases were found in cervical cancers with less common histologic subtypes. A worldwide prevalence study reported that 13.0% of squamous cell carcinomas and 28.2% of rarer cervical adenocarcinomas tested negative for HPV [29]. Based on their findings, the authors estimated that 3–4% of cervical cancers could be missed by routine HPV testing [29].

In our study, additional PCR-based testing of tumors from patients with confirmed primary cervical cancer after surgical staging showed that most samples with an initial HC2-negative test result (9/10) were HPV positive. Retesting showed that HC2-negative, HPV-positive results can be explained either by the failure of the initial test procedure to detect HR-HPV subtypes or by infection with other HPV subtypes not identified by the standard HC2 test. All established and validated HPV screening tests are designed to detect HR-HPV and will therefore miss the 1‒2% of cases associated with non-HR-HPV. This is an accepted gap of current routine HPV tests [34].

While the limitations of routine HC2 tests are well known, this study highlights the possibility of an erroneous diagnosis of primary cervical cancer based solely on biopsy. While the role of surgical staging remains controversial, this procedure was useful in this research context to fully characterize carcinomas invading the cervix. This study showed that HC2-negative tumors diagnosed as cervical cancer may originate from other primary sites. While secondary malignancies rarely occur in the uterine cervix, and mostly result from direct spread of other uterine tumors to the cervix, several primary solid tumors, including ovary, breast, stomach, gallbladder, pancreas, and lung, may to metastasize to the uterine cervix [35]. Similarly, lymphoma of the uterine cervix is rare, but may occur as a primary or secondary event in the disease course [36]. Other authors have previously highlighted the importance of making an accurate differential diagnosis before initiating therapy, particularly for patients with an atypical presentation of cervical adenocarcinoma [35], [36]. Our findings reinforce the recommendation that physicians should be aware of the possibility of cancers from other sites being misdiagnosed as primary cervical cancers and highlight the involvement of different HPV subtypes as potential causative agents. HPV subtypes not detected with the HC2 test can be identified using additional PCR-based tests as part of the differential diagnosis if clinically warranted.

This study has limitations inherent to all retrospective analyses of data collected from a single department. Nevertheless, all subjects were enrolled and evaluated according to a standard protocol. The median age of subjects included in the analysis was slightly younger than the overall average for cervical cancers overall (approximately 50 years). This minor difference can be explained by the exclusion of women with distant metastases at presentation and those with severe co-morbidity who were not able to undergo surgical staging; in addition, cancer cases from the colposcopy unit tend to be younger than the overall population of patients with cervical cancer. The study was initiated in 1999 and therefore includes subjects from a pre-vaccination era. The impact of HPV vaccination on the prevalence of HPV-negative cervical carcinomas is not yet known.

## 5 Conclusions

This study confirms the almost universal involvement of HPV infection in primary cervical cancer. HPV-negative tumors occurring in the uterine cervix are histologically distinct from true HPV-positive cervical cancer.
